# Linking genotype and phenotype in an economically viable propionic acid biosynthesis process

**DOI:** 10.1186/s13068-018-1222-9

**Published:** 2018-08-13

**Authors:** Carlos H. Luna-Flores, Chris C. Stowers, Brad M. Cox, Lars K. Nielsen, Esteban Marcellin

**Affiliations:** 1 Australian Institute for Bioengineering and Nanotechnology (AIBN), The University of Queensland, Brisbane, QLD 4072 Australia; 2 Queensland Node of Metabolomics Australia, The University of Queensland, Brisbane, QLD 4072 Australia; 3 BioEngineering and Bioprocessing R&D, Dow AgroSciences LLC, 9330 Zionsville Road, Indianapolis, IN 46268 USA

**Keywords:** Propionic acid, Propionibacterium, Genome shuffling, Genomics, Metabolomics, Transcriptomics

## Abstract

**Background:**

Propionic acid (PA) is used as a food preservative and increasingly, as a precursor for the synthesis of monomers. PA is produced mainly through hydrocarboxylation of ethylene, also known as the ‘oxo-process’; however, *Propionibacterium* species are promising biological PA producers natively producing PA as their main fermentation product. However, for fermentation to be competitive, a PA yield of at least 0.6 g/g is required.

**Results:**

A new strain able to reach the required yield was obtained using genome shuffling. To gain insight into the changes leading to the improved phenotype, the genome of the new strain was sequenced, and metabolomics and transcriptomics data were obtained. In combination, the data revealed three key mutations: (i) a mutation in the promoter region of a sugar transporter which enables an increase in the uptake rate of sucrose; (ii) a mutation in a polar amino acid transporter which improves consumption of amino acids and acid tolerance; and (iii) a mutation in a gene annotated as a cytochrome C biogenesis gene, which is likely responsible for the coupling of the Wood–Werkman cycle to ATP production were responsible for the phenotype. The bioprocess was further enhanced with a feeding strategy that achieved 70 g/L of product. The proposed bioprocess is expected to outperform the economics of the current ‘oxo-process’ by 2020.

**Conclusions:**

In this study, using genome shuffling, we obtained a strain capable of producing PA exceeding the commercial needs. The multiomics comparison between the novel strain and the wild type revealed overexpression of amino acid pathways, changes in sucrose transporters and an increased activity in the methylglyoxal and the glucuronate interconversion pathways. The analysis also suggests that a mutation in the cytochrome C biogenesis gene, coupled with ATP production through the Wood–Werkman cycle, may be responsible for the increased PA production.

**Electronic supplementary material:**

The online version of this article (10.1186/s13068-018-1222-9) contains supplementary material, which is available to authorized users.

## Background

Traditionally derived from fossil fuels, propionic acid (PA) is used in the food industry as a preservative. Due to its three-carbon backbone, PA has found increased use for the synthesis of monomers and polymers. As a result, the market for PA has steeply grown 5.1% per annum to over 350,000 tonnes/year over the past decade. This increased demand results in the need for new PA infrastructure and more sustainable production routes [1]. Bacterial fermentation addresses many environmental concerns and offers a sustainable alternative for its production. *Propionibacteria* naturally produce PA as their main fermentation product using the Wood–Werkman cycle [2, 3]. Recent economic and ecological assessment called for a yield of 0.6 g/g for an economically viable scalable process [1, 4].

PA yield determines economic viability as the feedstock and the downstream purification process contributes largely to the production cost of PA biosynthesis [1]. Given limitations in the availability of tools for rational strain design, random mutagenesis and/or adaptive evolution have been the best strategies to improve the yield of PA in *propionibacterium*. We have previously used genome shuffling (GS) to obtain a recombinant strain capable of producing PA at a yield of 0.55 g/g which was close to the target yield of 0.6 g/g [5–7]. Developed in the early 2000s, GS is a rapid phenotypic improvement technique based on protoplast fusion [8, 9]. GS relies on multiple combinatorial recombination events in well-conserved regions of the genomes [5] of the parental strains leading to the desired phenotype. The technique has recently been reviewed in detail by [10]. Success heavily depends on finding appropriate screening methods to select for the superior phenotype.

In *Propionibacterium*, PA biosynthesis is closely linked to energetics. The Wood–Werkman cycle is involved in generating ATP, and balancing redox [11]. As a key biosynthetic pathway of energy metabolism, we were able to correlate PA production with faster growth rate. At the same time, faster growth rate is generally influence by the more efficient energetics. Cells regulate their metabolism through intracellular concentration of energy-linked metabolites. For example, imbalanced AMP, ADP, ATP, cAMP and c-di-AMP trigger mutations in the central carbon metabolism [12]. Such example shows that in response to a metabolic imbalance, cells respond by upregulating alternative pathways. One example of such a pathway is the methylglyoxal pathway which is used to regulate the concentration of imbalanced metabolites [13]. For example, in *E. coli*, induction of the methylglyoxal pathway has been observed as a response for glucose-6-phosphate deregulation [14–16]. Methylglyoxal is produced from dihydroxyacetone phosphate and can be induced by high concentrations of the latter. Reports suggest that the methylglyoxal pathway relieves cells from stress by elevating levels of sugar phosphates [17].

The last decade has also seen rapid advances in high-throughput analytics for strain characterization [18], opening new avenues for understand genotypic changes leading to improved phenotypes. Here, we used a systematic approach to understand the genomic changes leading to a commercially viable process for PA production. The new strain obtained by genome shuffling was obtained from a combination of various strains of *Propionibacteria* [5]. Genomics, metabolomics and transcriptomics were then used to understand the mutations leading to the improved phenotype. A systematic molecular characterization of the phenotype revealed three key mutations. The analysis was complemented with the design of a non-structured growth-model [19, 20]. The model was used to design a feeding strategy resulting in 70 g/L of PA, enabling further reduction of downstream purification costs. The process was economically assessed using a revised economic model to predict if the current biological process will be feasible at a 170 kta (thousand tons per year) scale.

## Methods

### Strains

To access genomic diversity, genome shuffling (GS) was performed using *P. acidipropionici* F3E8 (previously obtained using GS and the wild-type strains *P. acidipropionici* ATCC 55737 and *P. acidipropionici* ATCC 4875 [5]), *P. acidipropionici* ATCC 4875, *P. acidipropionici* ATCC 4965, *P. intermedium* ATCC 14072 and *P. jensenii* ATCC 9617. Wild-type strains were selected from a collection of 17 strains previously characterized on sucrose [7]. All strains were kept at − 80 °C using glycerol (20%) as cryoprotectant.

### Media

Except when otherwise specified, the PAM medium used contained yeast extract (10 g/L), trypticase soy (5 g/L), K_2_HPO_4_ (0.05 g/L), MnSO_4_ (0.05 g/L), and sucrose (40 g/L). Agar (15 g/L) was added to PAM plates. 80 g/L instead of 40 g/L was used in instrumented fermenters for batch cultures. The feeding solution of fed-batch cultures consisted in PAM media fivefold concentrated and a sugar concentration of 350 g/L. Sucrose was always autoclaved separately. The chemically defined medium (CDM) contained: sucrose (20,000 mg/L), FeSO_4_·7H_2_O (10 mg/L), Fe(NO_3_)_2_·9H_2_O (1 mg/L), K_2_HPO_4_ (100 mg/L), KH_2_PO_4_ (500 mg/L), MgSO_4_·7H_2_O (500 mg/L), MnSO_4_ (10 mg/L), CaCl_2_·6H_2_O (10 mg/L), NaH_2_PO_4_·H_2_0 (1597.5 mg/L), CoCl_2_·6H_2_O (10 mg/L), Na_2_HPO_4_ (3675 mg/L), biotin (0.2 mg/L), riboflavin (2 mg/L), thiamine hydrochloride (1 mg/L), vitamin B12 (0.2 mg/L), and pantothenic acid (2 mg/L) and 12 amino acids. The 12 amino acids used were arginine (200 mg/L), asparagine (2000 mg/L), cysteine (200 mg/L), glutamine (200 mg/L), histidine (200 mg/L), leucine (200 mg/L), methionine (200 mg/L), phenylalanine (200 mg/L), proline (200 mg/L), serine (200 mg/L), tryptophan (200 mg/L) and tyrosine (200 mg/L).

### pH gradient plates

The PA/pH gradient plates were prepared using PAM agar supplemented with 5 g/L of PA salt adjusted to pH = 3 or pH = 6.5 using square plates (100 × 100 mm). The plates were prepared by lifting the plates 0.5 cm and by pouring the neutral pH agar first. Once the first layer was solidified, the plate was placed horizontally, and the PAM agar at low pH was added. Plates were incubated overnight at room temperature to allow for the formation of the pH gradient. The pH gradient was confirmed with pH strips indicators.

### Protoplast formation, fusion, and regeneration

GS was performed as described in our previous study [5]. Protoplast formation buffer (PFB) was made from (g/L): sodium succinate (40.5), sucrose (42.75), and MgCl_2_ (1.9). PFB was dissolved in one litre of Tris–HCl 0.05 mol/L at pH 7.1. Regeneration buffer (RB) was made from (g/L): yeast extract (10), trypticase soy (5), KH_2_PO_4_ (1.5), K_2_HPO_4_ (2.5), and BSA (5). The media were adjusted to pH 7. For the protoplast preparation, cells were grown for 24 h in PAM media supplemented with 40 g/L of glucose and 1% of glycine. Cells were then conditioned in PAM media containing 1% of glycine and 120 g/L of glucose for an extra 24 h. After at least ten generations, cells were washed twice using PBS and fixed to an A600 of 0.2 in a lysozyme solution containing 15 mg/mL (600,000 U/mL) in PFB. Cell walls were digested in a 125 mL flask for two h at 120 rpm and 40 °C. Protoplasts were detected in a light microscope using the 100× oil immersion objective and counted using a haemocytometer. When appropriate, protoplasts were regenerated in RB (pH 7 for 48 h at 32 °C). For the protoplast fusion, to motivate recombination, protoplasts were treated with UV light (36 W at 253.7 nm to a distance of 30 cm) for 0.5 min or were treated with heat at 60 °C for 2 h. Cells were mixed, centrifuged and re-suspended in 500 µL of PFB. Then, 500 µL of PEG 6000 (80%) with 20 mmol/L CaCl_2_ was added. Fusion conditions were pH 7.1, time 30 min, and temperature 32 °C. After fusion, 5 mL of PFB was added and centrifuged at 3500 rpm for 5 min. Protoplasts were washed twice with 5 mL of PFB and re-suspended in 1 mL of RB.

### GS and strain selection

Strain diversity was created by shuffling the wild-type strains *P. acidipropionici* ATCC 4875, *P. acidipropionici* ATCC 4965, *P. intermedium* ATCC 14072, and *P. jensenii* ATCC 9617. Separately, the strain *P. acidipropionici* 4875 was shuffled with the other three wild-type *Propionibacterium* strains. Cells were selected from the acidic side of pH/PA gradient plates. In total, three rounds of genome shuffling were performed with each set of strains. Next, another three rounds of GS were performed with the two libraries of strains, the parental strains *P. acidipropionici* ATCC 4875, *P. acidipropionici* ATCC 4965, *P. intermedium* ATCC 14072, *P. jensenii* ATCC 9617, and the mutant *P. acidipropionici* F3E8. Finally, the new recombinants were isolated by serial dilutions in PAM media agar plates. Plates were incubated in anaerobic jars containing AnaeroGen sachets. Oxoid strip anaerobic indicators were used to confirm anaerobiosis. Individual recombinants were randomly selected and screened in a 96-well plate containing 100 µL of PAM media at pH 5 and 25 g/L of PA. Growth was monitored using a micro-plate reader (FLUOStar Omega, BMG Labtech, Mornington, Victoria, Australia) adapted to maintain anaerobic conditions through a continuous injection of nitrogen. The selection criteria were based on an acid tolerance improvement which was determined by an acidic ratio comparison between the new strains and the wild type—ratios were calculated by dividing the specific growth rate under acidic conditions over specific growth rate under non-acidic conditions. The best performing strains (Additional file 1: Table S1) were scaled up to 250 mL serum bottles with a working volume of 100 mL. Serum bottles were incubated using an orbital shaker incubator (Multitron, Infors-HT, Bottmingen, Switzerland) at an agitation rate of 100 rpm (2.5 cm orbit) and a working temperature at 32 °C for 96 h.

### Bioreactor fermentations

Fermentations were performed using 2-L Applikon fermenters with a working volume of 1 L for batch cultures. Fermenters were equipped with probes and controllers for pH, dissolved oxygen, temperature, and agitation. The agitation rate was controlled with two Rushton impellers at 300 rpm. The pH was controlled at 6.5 using 10 M NaOH. The temperature of the culture was maintained at 32 °C using an electric jacket. Before inoculation, the fermenters were sparged with N_2_ for at least 15 min. A constant head space N_2_ flow was kept for the entire fermentation at a flow rate of 0.3 L/min. Cultures were activated under sterile conditions in a 1.5 mL Eppendorf tubes containing 1 mL of PAM media inoculated with 0.8% (v/v) of a glycerol stock. Inoculums were grown for 24 h at 32 °C. Cultures were transferred into a 15 mL Falcon tube containing 14 mL of PAM media and allowed to grow for 24 h. 5% (v/v) of this culture was used to inoculate 250 mL serum bottles containing 100 mL of PAM media and allowed to grow for an additional 24 h. Cells from the serum bottles growing in mid-exponential phase were used to inoculate fermenters at an initial A600 of 0.3. Fed-batch cultures were performed using the same culture conditions at a working volume of 0.7 L.

### Model development for fed-batch design

The construction of the batch and fed-batch models was adapted from [19]. The model was first developed as a batch culture and extrapolated to a fed-batch system. The parameters of the batch model were obtained from the fermentation in PAM medium using 80 g/L of initial sucrose concentration. The following assumptions were made:

1. Sucrose is the only limiting carbon source.
2. There is no nitrogen limitation.
3. PA and acetic acid (AA) are the only inhibitory metabolites.
4. The pH is known and controlled throughout the fermentation at pH = 6.5.

#### Batch model

The differential mass balance Eqs. (1)–(8) describe the dynamic of propionic acid production in batch fermentation as follows:

1 $$\frac{{{\text{d}}X}}{\text{dt}} = uX\quad \left( {\text{biomass}} \right)$$

2 $$\frac{{{\text{d}}S}}{\text{dt}} = - \;{\text{qs}}X\quad \left( {\text{sucrose}} \right)$$

3 $$\frac{{\text{dPA}}}{{\text{dt}}} = \left[ {K1u + \beta_{\text{pa}} } \right]X\quad \left( {\text{propionic acid}} \right)$$

4 $$\frac{{\text{dPYR}}}{{\text{dt}}} = \left[ {K2u + \beta_{\text{pyr}} - K3 \frac{\text{PYR}}{{{\text{PYR}} + K_{\text{pyr}} }}} \right]X\quad \left( {\text{pyruvic acid}} \right)$$

5 $$\frac{{\text{dAA}}}{{\text{dt}}} = \left[ {K4 u + \beta_{\text{aa}} + K5\frac{\text{PYR}}{{{\text{PYR}} + K_{\text{pyr}} }}} \right]X\quad \left( {\text{acetic acid}} \right)$$

6 $$\frac{{\text{dSA}}}{{\text{dt}}} = \left[ {K6 u + \beta_{\text{sa}} + K7\frac{\text{PYR}}{{{\text{PYR}} + K_{\text{pyr}} }}} \right]X\quad \left( {\text{succinic acid}} \right)$$

7 $${\text{qs}} = {{\text{rs}}}_{{\text{max}}} \left( {\frac{S}{{K_{{\text{s}}} + S}}} \right)\left( {\frac{{\text{kipa}}}{{{{\text{kipa}}} + {{\text{PA}}}}}} \right)\left( {\frac{{\text{kiaa}}}{{{{\text{kiaa}}} + {{\text{AA}}}}}} \right)\;\left( {{\text{specific substrate consumption}}} \right)$$

8 $$u = \left( {{\text{qs }}Y_{\text{xs}} } \right) - \left( {{\text{ms}} Y_{\text{xs}} } \right)\quad \left( {\text{specific growth rate}} \right)$$

Equation (1) represents the growth rate and Eq. (8) its specific rate (*µ*). The latter equation was adapted from the Leudking and Piret expression. Equation (2) represents the consumption rate of sucrose and Eq. (7) represents its specific rate which considers inhibition by PA and AA. PA, pyruvate (PYR), AA, and succinic acid (SA) production rates are represented by Eqs. (3), (4), (5), and (6), respectively; they consider growth associated (*K*) and non-growth associated production parameters (*β*). The Eqs. (5) and (6) also describe the consumption of pyruvate generated by Eq. (4) to produce AA, and SA, respectively.

#### Fed-batch model

The batch model was extrapolated to a fed-batch culture to design the feeding profile. High PA production and sugar accumulation below 40 g/L were the criteria to design parameters for the fed-batch feeding strategy. The fed-batch model was represented with Eqs. (9)–(16). To obtain these equations, a differential equation to represent volume (*V*) and calculate the factor dilution (*D*) were added to the Eqs. (1)–(6).

9 $$\frac{{{\text{d}}x}}{\text{dt}} = uX - DX\quad \left( {\text{biomass}} \right)$$

10 $$\frac{{{\text{d}}S}}{\text{dt}} = - \;{\text{qs}}X + D\left( {S{\text{o}} - S} \right)\quad \left( {\text{sucrose}} \right)$$

11 $$\frac{{\text{dPA}}}{{\text{dt}}} = \left[ {K1 u + \beta_{\text{pa}} } \right]X - D{\text{PA}}\quad \left( {\text{propionic acid}} \right)$$

12 $$\frac{{\text{dPYR}}}{{\text{dt}}} = \left[ {K2 u + \beta_{\text{pyr}} - K3 \frac{\text{PYR}}{{{\text{PYR}} + K_{\text{pyr}} }}} \right]X - D{\text{PYR}}\quad \left( {\text{pyruvic acid}} \right)$$

13 $$\frac{{\text{dAA}}}{{\text{dt}}} = \left[ {K4 u + \beta_{\text{aa}} + K5\frac{\text{PYR}}{{{\text{PYR}} + K{\text{pyr}}}}} \right]X - D{\text{AA}} \quad \left( {\text{acetic acid}} \right)$$

14 $$\frac{{\text{dSA}}}{{\text{dt}}} = \left[ {K6 u + \beta_{\text{sa}} + K7\frac{\text{PYR}}{{{\text{PYR}} + K_{\text{pyr}} }}} \right]X - D{\text{SA}}\quad \left( {\text{succinic acid}} \right)$$

15 $$\frac{{{\text{d}}V}}{\text{dt}} = F \quad \left( {\text{flow rate}} \right)$$

16 $$D = {F \mathord{\left/ {\vphantom {F V}} \right. \kern-0pt} V}\quad \left( {\text{factor dilution}} \right)$$

#### Reliability of the model

The coefficient of determination (*R*^2^) was used to determine the reliability of the model. The *R*^2^ was calculated as follows:

$$R^{2} = \frac{1}{m}\mathop \sum \limits_{j = 1}^{m} \left( {1 - \frac{{\text{SSE}}}{{\text{SST}}}} \right)$$

$${\text{SSE}} = \mathop \sum \limits_{i = 1}^{n} \Delta_{i}^{2}$$

$${\text{SST}} = \mathop \sum \limits_{i = 1}^{n} (y_{i} - \bar{y})^{2}$$

$$\bar{y} = \frac{1}{n}\mathop \sum \limits_{i = 1}^{n} y_{i}$$

#### Parameters estimation

Excluding the *K*_s_ value, the package SBPDgui of the System Biology Toolbox 2 (SBTOOLBOX2) was used to determine the parameters of the model [21]. The *K*_s_ value of Eq. 7 was determined experimentally using data from serum bottle fermentations in PAM medium at different concentrations of sucrose.

### Calculation of fermentation parameters

Specific growth rate (*µ*) was calculated at the mid-exponential phase. Specific growth rate calculations in 96-well plates were performed using the program GrowthRates [22]. For consistency, volumetric productivity (*P*_v_) was calculated for the same time interval (ranging from 15 to 30 h). Yield (*Y*_ps_) was calculated using the total PA produced over the consumed substrate. PA:AA and PA:SA ratios were calculated using total organic acid production. The PA:AA and PA:SA ratios are indication of efficiency, in which the higher the ratios the lower is the production of the by-products AA and SA, respectively. The specific consumption rate of sucrose (*qs*) and the specific production rate of PA (*qp*) were computed at mid-exponential phase multiplying specific growth rate by the linear correlations of sugar or PA with biomass.

### Intracellular pH measurement (pHi)

Intracellular pH (pHi) was measured using the method adapted from [23]. Fluorescence of 2′,7′-bis-(2-carboxyethyl)-5(and 6)-carboxyfluorescein acetoxymethyl ester (BCECF AM) was used to measure pHi. BCECF AM is a pH-sensitive fluorescein derivative probe with a dual-excitation ratio. Briefly, cells (2 mL with a A600 of 2) were centrifuged (12,000 rpm, 1 min) and washed with 50 mM HEPES-K buffer (pH 8). The pellet was re-suspended in 2 mL of the same buffer and incubated with 1 μL of 1 μM BCECF AM for 20 min at 32 °C. After, cells were washed with 50 mM potassium phosphate buffer (pH 7). The pellet was re-suspended in the same buffer and 50% of the suspension was filtered. Fluorescence intensities were performed in a fluorescence spectrophotometer with an excitation spectrum of 490 nm (pH sensitive) and 440 nm (pH-insensitive). The emission was at 535 nm. The ratio of the emission intensity at 490 and 440 of both the suspension (*S*) and filtrate (*F*) was determined as follows: *R* = (*S*_490_ − *S*_440_)/(*F*_490_ − *F*_440_). This ratio and a calibration curve were used to calculate the pHi.

The calibration curve was determined for each strain as follows. Valinomycin and nigericin (Sigma) were added to each strain (A600 of 2) to a final concentration of 50 µM to maintain equilibration of pHi with extracellular pH (pHex). The cultures were then incubated at 32 °C for 20 min. Cells (2 mL with an A600 of 2) were centrifuged, washed and re-suspended in 2 mL of buffer at pH 4, 5, 6, 7, or 8 (50 mM citrate buffer, pH 4 and 5; 50 mM phosphate buffer, pH 6, 7 and 8). After, 1 µL of 1 µM BCECF AM was added and incubated for 20 min at 32 °C. After the incubation, cells were washed and re-suspended using the respective buffer. Finally, 50% of the suspension was filtered, and the fluorescence determination and ratio calculation were performed as mentioned above.

### Analytics

The absorbance of the culture was measured at 600 nm using a Biochrom Libra S12 UV/Vis spectrophotometer. Organic acids, carbohydrates, and alcohol were quantified by ion-exclusion chromatography as described elsewhere [24].

### DNA sequencing, de novo assembly, annotation, and comparison

Genomic DNA was extracted using PureLink Genomic DNA Mini kit (Invitrogen Cat. No. K1820-01) and quantified using Nanodrop 1000 (Thermo Scientific) and Qubit dsDNA BR assay kit (Life Technologies Cat. No. Q32850). The quality of the DNA was determined by running a 1% agarose gel with DNA gel stain SYBR safe (Life Technologies Cat. No. S33102). The gel was visualized with a ChemiDoc MP system (Bio-Rad). The PacBio sequencing platform was used to obtain the complete genome of the new recombinant strain. The PacBio library preparation was performed using the protocol for 20 Kb selected with the BluePippin system. The sequencing chemistry was the release P6-C4 by PacBio and loaded by magnetic beads. The genome assembly was performed with the SMRT portal. This portal was also used to align the reads and call the variants of the sequenced genome. Finally, the RAST and SEED viewer servers were used to annotate and visualize the assembled genome, respectively [25, 26]. Genome-to-genome distance calculator was used to determine similarity between two whole genome sequences [27]. Mauve was used to align multiple genome sequences and trace back genomic mutations [28]. Blast was used to extract specific genomic regions [29]. The genome was submitted to NCBI and assigned the accession number GenBank CP031057.

### Intracellular metabolites extraction

Cells sampled at mid-exponential phase were used for intracellular metabolomics analyses as described in [30–33]. Metabolites extraction was performed with 50% acetonitrile (ACN). Briefly, 1–20 ODs (1 OD = 1 mL of a culture with an OD of 1) were harvested and centrifuged at 20,172*g* for 2 min at room temperature. The supernatant was then discarded, and the pellet was resuspended in 50% ACN. This solution was vortexed for 10 s every 2 min for three times and centrifuged for 3 min at 4 °C at 20,172*g*. After, the supernatant was placed into a tube and frozen at − 80 °C before being freeze dried. Finally, the powder was resuspended in 0.5 mL of MilliQ water. Intracellular metabolites of the central carbon metabolism were analysed by LC–MS and intracellular amino acids by HPLC (method described above). Metabolite concentrations were standardized using dry cell weight values. The factor to convert A600 to dry cell weight (g/L) was 0.29 for *P. acidipropionici* ATCC 55737 and 0.25 for *P. acidipropionici* WGS7.

### Statistical analyses of intracellular metabolomics

Metabolomics data were normalized and analysed for statistical significance using the R Package “metabolomics” [34].

### RNA extraction, sequencing, and analyses

Cells sampled at mid-exponential phase were used for RNA extraction as described in [35]. Briefly, 50 ODs (1 OD = 1 mL of a culture with an OD of 1) were harvested and centrifuged at 4000*g* for 10 min at room temperature. The supernatant was removed, and 5 mL of RNA later reagent was added to the pellet. After 8–24 h of incubation at 4 °C, the RNA later was removed by centrifugation and the pellet stored at − 80 °C for further use. RNeasy^®^ Mini Kit (Qiagen) was used to extract the RNA and the RNA Clean and Concentrator-25 Kit (Zymo) to clean it. Next, the RNA was enriched depleting the ribosomal RNA with the Ribo-Zero Magnetic Kit (Illumina). The samples were cleaned and concentrated with the RNA Clean and Concentrator-5 Kit (Zymo). The quality of the RNA was evaluated by a Bioanalyzer. Finally, the samples were sequenced using the Illumina platform 100 bp Pair End. Tophat, Cufflinks, and CuffDiff were used to align the RNA-seq reads against the reference genome *P. acidipropionici* ATCC 55737 [36], normalize and annotate the transcripts, and evaluate the differential expression, respectively [37]. The cutoff for significant differentially expressed genes was twofold change and *q* < 0.05. The raw transcriptomic data and complete differential expression list (also found in Additional file 2) were deposited in the Gene Expression Omnibus data repository under the accession number GSE86950.

## Results and discussion

**Fig. 1:**
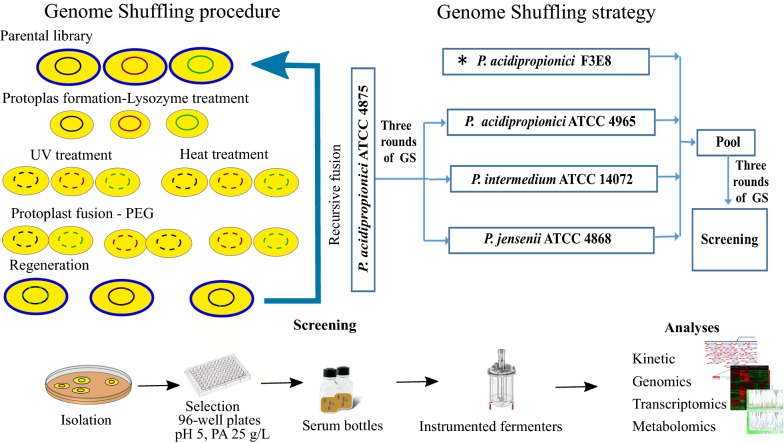
The general procedure to obtain and analyse WGS7. Left: genome shuffling procedure. Right: genome shuffling strategy. Bottom: screening procedure and analyses. PEG: polyethylene glycol. (Asterisk) *P. acidipropionici* strain previously obtained in [5]

*Propionibacterium* fermentation is inherently inhibited by acid accumulation that ultimately results in growth arrest [38]. Previously, immobilized cells and extractive fermentation systems have been used to improve production with limited success [6, 39–42]. Due to scalability problems using immobilized bed reactors, the best alternative is to develop an improved biocatalyst able to tolerate acidic conditions. Such phenotypes can be obtained using GS. We recently obtained a strain capable of producing PA with a yield of 0.55 g/g of glucose using GS [5]. As part of that study, ten strains obtained through GS were sequenced. The analysis showed a correlation between the improved phenotype and acid tolerance. Therefore, to further enhance acid tolerance, we used GS and a PA/pH gradient strategy for screening for higher producers. Firstly, we fused *P. acidipropionici* ATCC 4875 with *P. acidipropionici* ATCC 4965, *P. intermedium* ATCC 14072, and *P. jensenii* ATCC 9617. The strains obtained were then fused again with our previously obtained mutant strain *P. acidipropionici* F3E8 (Fig. 1) [5].

Strains were then characterized using 96-well plates containing 25 g/L of PA and a pH of 5. The screening resulted in 13 candidates that were grown in serum bottles (Additional file 1: Table S1). Six out of the thirteen strains had a higher PA yield relative to F3E8. All six strains grew to a higher cell density. The top-three producing strains were moved to instrumented fermenters, and the best strain was selected (hereafter named WGS7). The strain was characterized using a multiomics approach which has been shown to help elucidating links between phenotype and genotype.

**Fig. 2:**
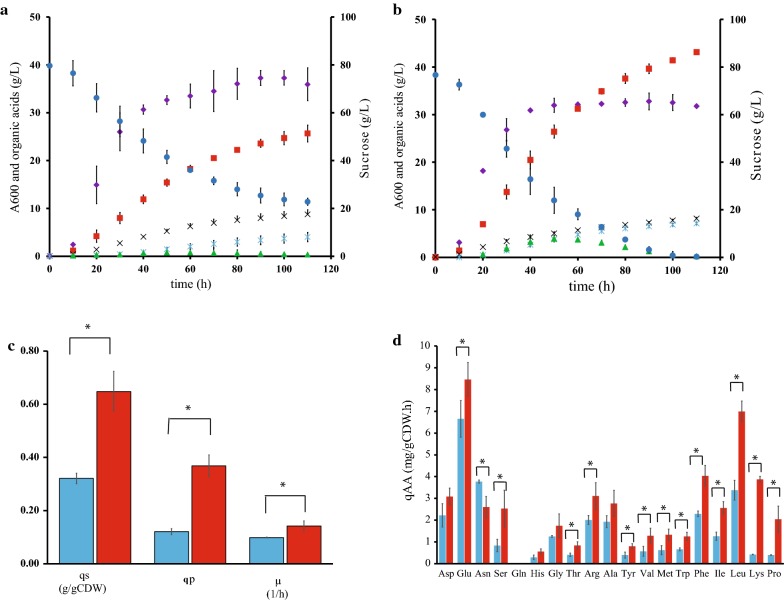
Fermentation profile in 2-L fermenters for the closest parental strain, *P. acidipropionici* ATCC 55737, and the new strain obtained through genome shuffling, *P. acidipropionici* WGS7. **a** Fermentation profile for *P. acidipropionici* ATCC 55737, **b** fermentation profile for *P. acidipropionici* WGS7. Absorbance: purple; propionic acid: red; glucose: blue; succinic acid: light blue; acetic acid: black; pyruvate: green. **c** Specific consumption rate of sucrose (*qs*), specific production rate of PA (*qp*), and the specific growth rate (*µ*). **d** Specific consumption rates of the free amino acids presented in the PAM medium. Light blue bars: *P. acidipropionici* ATCC 55737. Red bars: *P. acidipropionici* WGS.7. The data represent the average of two biological replicates for each strain. The specific rates were calculated in the mid-exponential phase

**Fig. 3:**
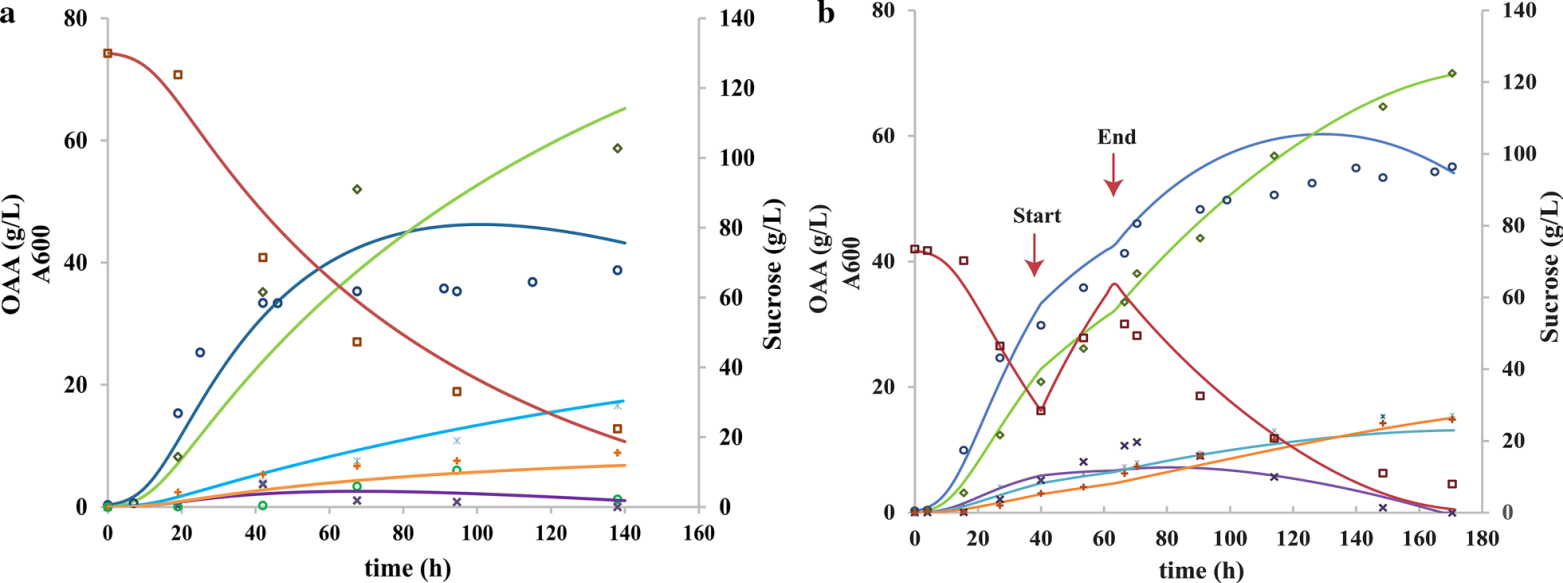
Fermentation profile of 2-L bioreactors with *P. acidipropionici* WGS7. **a** Batch culture with 130 g/L of sucrose. **b** Fed-batch culture with a feeding solution of 5× the PAM media and a constant feeding rate of 0.0066 L/h from time 40 h (start) to 64 h (end). For both figures: continuous line indicates model simulation and markers indicate experimental data. Absorbance: blue; propionic acid: light green; sucrose: red; succinic acid: light blue; acetic acid: orange; pyruvate: purple; lactate: green. Experimental absorbance: blue circle; experimental propionic acid: light green lozenge; experimental sucrose: red square; experimental succinic acid: light blue asterisk; experimental acetic acid: orange plus sign; experimental pyruvate: black multiplication sign. oaa: organic acids; experimental lactic acid: light green circle. No simulation data are shown for lactic acid

As shown in Table 1 and in Fig. 2, WGS7 had an improvement of 37%, 85%, 43%, and 80% in the PA yield, PA:AA ratio, growth rate, and volumetric productivity, respectively. The PA:AA improvement was the main contributor for the reduction in cost of the PA downstream purification in WGS7 [1]. The PA:SA ratio was unchanged (*p* > 0.05). We also observed an improvement in the intracellular pH gradient (ΔpH = internal pH − extracellular pH). The strain WGS7 had a ΔpH = 1.27 compared to the wild type which had a ΔpH = 0.43 (Table 1). Improvements in ΔpH have been reported to improve acid tolerance in other *Propionibacterium* strains [23]. A feeding profile was then used to increase PA production, designed using a non-structured model. The feeding strategy was designed to keep sucrose concentrations below 40 g/L in order to maintain the high PA yields observed in optimal batch conditions (Figs. 2b and 3b, respectively). The fermentation data presented in Fig. 2b were used to determine the parameters for the mathematical model (Additional file 3: Table S5). The comparison between the experimental data and the simulation for fed-batch culture had a *R*^2^ of 0.96 (Fig. 3b), suggesting a high accuracy of the model to predict the production of PA in WGS7. The *R*^2^ for the batch culture at high initial sugar concentration (130 g/L) was 0.90 (Fig. 3a). This value suggests issues in the model to accurately predict PA at high sugar concentrations. In the fed-batch culture, WGS7 had a 58% increase in PA production compared to its optimal PA production in batch culture (Figs. 2b, 3b, and Additional file 3: Table S6). The WGS7 fed-batch culture, producing 70 g/L of PA, enables for the first time, an economically viable bioprocess using *Propionibacterium* spp.

**Table 1:** Parameters for *P. acidipropionici* ATCC 55737 and *P. acidipropionici* WGS7 grown in 2-L fermenters

Strain	^+^Yps (g/g)	PA:SA (g/g)	^+^PA:AA (g/g)	^a+^*P*_v_ (g/L h)	^+^∆pH	^+^Final PA (g/L)
*P. acidipropionici* ATCC 55737	0.45 ± 0.03	6.28 ± 0.94	2.95 ± 0.35	0.530 ± 0.21	0.43 ± 0.04	26.28 ± 1.88
*P. acidipropionici* WGS7	0.62 ± 0.01	6.19 ± 0.23	5.45 ± 0.41	0.955 ± 0.15	1.27 ± 0.10	44.21 ± 0.93

∆pH: pHi − pHext (pHi: internal pH; pHext: external pH)

^a^Calculated during the range of time: 15–30 h

^+^*p* value < 0.05

### Comparative genomics

To evaluate similarities between the strains used for GS, we used the genome-to-genome distance calculator [27]. As expected, high genomic similarities were found between the *P. acidipropionici* strains with a similitude of 86.86% ± 3.58%, which corresponded to a genomic distance of 0.016 ± 0.004. Comparatively, the similarity between *P. intermedium* ATCC 14072 and *P. jensenii* ATCC 9617 was 90.70%, which corresponds to a genomic distance of 0.010. On the other hand, the similarity between the *P. acidipropionici* strains and the other *Propionibacterium* spp. was only 26.83% ± 1.21%, which corresponded to a genomic distance of 0.163 ± 0.006.

The best strain obtained using GS, WGS7, was sequenced using PacBio RS II and assembled using the SMRT Portal. The 3.16 Mb genome was annotated using RAST [25], which found 3333 CDS and 65 RNAs. Using the SMRT portal, the closest relative found was *P. acidipropionici* ATCC 55737 (hereafter denoted ATCC55737). As such, ATCC55737 was used for comparison hereafter. The genomic comparison found 17 SNPs and seven INDELs (Additional file 4: Figure S2 and Additional file 5: Table S3). The genomic regions with mutation in WGS7 were aligned with the other strains used for GS. The comparison suggests that two mutations in WGS7 came from the wild-type strains used for GS. The mutation G1917729A was also found in *P. acidipropionici* ATCC4875 and *P. acidipropionici* ATCC4965, and the mutation A3335969G was found in all wild-type strains used for GS (Additional file 6: Figures S4, S5). The other mutations, found in WGS7, could not be linked to any of the other strains and, therefore, their origin remains elusive. It is possible that the other mutations are coming from adaptive laboratory evolution in response to the acid challenge, which is a common technique routinely used in industry and academia for strain improvement [38].

### Linking metabolomics

**Fig. 4:**
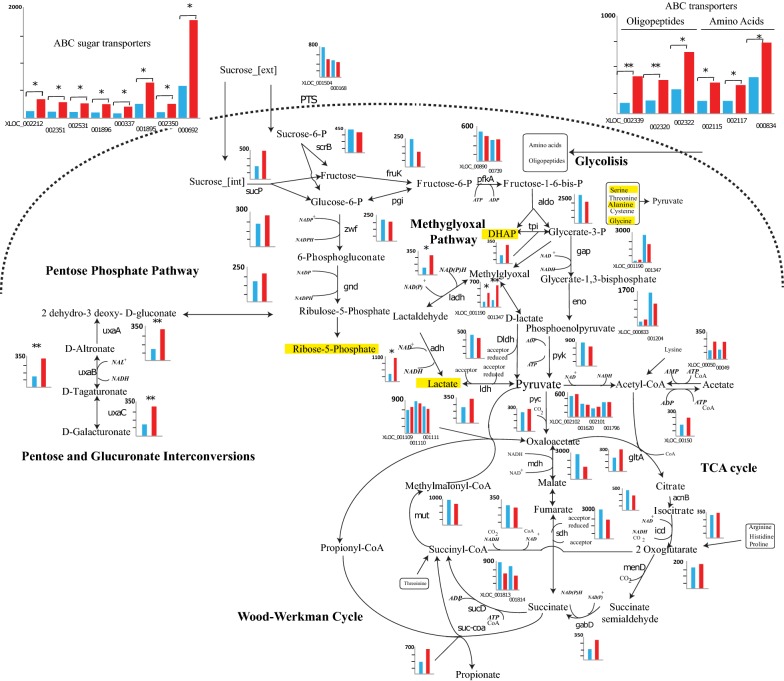
Metabolic pathways representation of the central carbon metabolism of *P. acidipropionici*. Bar charts represent RNA-seq data in FPKM. Light blue bars are for *P. acidipropionici* ATCC 55737 and red bars for *P. acidipropionici* WGS7. Significance from the Cufflink analysis is indicated with asterisk, where *q < 0.05, **q < 0.01. Metabolites in yellow were found with an increased concentration in WGS7 (*p* < 0.05). For clarity, the gene IDs name can be found in Additional file 9: Table S4

To better understand the links between phenotype and genotype, a systems-level characterization, previously shown to be a useful tool to characterize links between genotype and phenotype [23, 39, 40], was performed. Extracellular metabolites were measured across the fermentation time course (Fig. 2). The growth rate, sucrose, amino acids uptake rates and the PA production rate were calculated from the exponential growth phase. WGS7 had a 96% higher sucrose uptake rate and a 216% higher specific PA production rate (Fig. 2c) compared to ATCC55737. All the sugar was depleted in WGS7 within 100 h, whereas ATCC55737 only consumed 69% of the total sugar provided. A mutation, G1917729A, was found in the promoter region of an ABC sugar transporter (Table 2). Mutation G1917729A in WGS7 seems to come from either *P. acidipropionici* ATCC4875 or *P. acidipropionici* ATCC4965 (Additional file 6: Figure S4). This gene was found overexpressed (log_2_ 2.2, *q* < 0.05) in the transcriptomics data (Fig. 4). In agreement with the data, the sucrose uptake rate was increased in WGS7 (Fig. 2c). Upregulation of sugar transporters is commonly used to enhance production in metabolic engineering. Many studies have previously overexpressed sugar transporters to enhance production. For example, transporters have been overexpressed in *E. coli* to improve sugar consumption and enhance production of several metabolites [43, 44].

**Table 2:** Mutations selected in this study to understand high PA production in WGS7

Genome coordinate^a^	Type	Reference	Alternate	Gene function	Origin	Remark
1,917,729	SNP	G	A	Multiple sugar ABC transporter, substrate-binding protein	ATCC4965 or ATCC4875	Promoter
1,487,806	SNP	C	T	Cytochrome c-type biogenesis	E	R134Q
2,293,187	SNP	C	T	Amino acid ABC transporter	E	Silent

*SNP* single nucleotide polymorphism, *E* elusive

^a^Genome coordinate in *P. acidipropionici* ATCC 55737. ATCC4965 *P. acidipropionici* ATCC 4875; ATCC4875 *P. acidipropionici* ATCC 4965. Silent: no change in amino acid

Sixty-four intracellular metabolites, extracted in mid-exponential phase, were analysed by LC–MS or HPLC. Six intracellular metabolite concentrations were found to be statistically significant (*p *<* 0.05*) using the R Package “metabolomics” between the strains compared. From these significant intracellular metabolites, the concentration for serine (Ser) and glycine (Gly) was decreased. The concentrations for alanine (Ala), dihydroxyacetone phosphate (DHAP), ribulose-5-phosphate (R5P) and lactate (Lac) were increased (Fig. 4; Additional file 7: Figure S1 and Table S2).

The genomic analysis found the mutation C2293187T in an ABC polar amino acid transporter (Table 2). Polar amino acids include arginine, lysine, aspartate, glutamine, asparagine, glutamate, serine, threonine, tyrosine, cysteine, methionine, and tryptophan. All those amino acids, except for aspartate and asparagine, were consumed faster in WGS7 than in the wild type (Fig. 2d). Several other investigations have suggested that amino acids metabolism can be closely linked to acid tolerance mechanisms [23]. Here, we observed an improvement in intracellular pH in WGS7 compared to the wild type. In agreement with that observation, arginine, glutamate, lysine, and serine were consumed faster in the complex medium in the WGS7 strain (Fig. 2d). To test their involvement in acid tolerance, each amino acid was added to the CDM separately in both strains. The addition of arginine increased 13% the final PA titre in WGS7 compared to only 3% in the wild type (Additional file 8: Figure S3). Arginine is metabolized through the arginine deiminase (ADI) pathway in *P. acidipropionici.* Other investigators have reported that overexpression of the arginine deiminase pathway increased PA production by tenfold in *P. jensenii* [45]. However, RNA-seq data showed that the arginine/ornithine antiporter gene was downregulated in WGS7 and that the pathway is not differentially transcribed (Additional file 2). Downregulation suggests that the ADI pathway is unlikely to be involved in acid tolerance in the new strain and that the polar amino acid transporter is likely to be transporting a different amino acid. Lysine consumption rate in WGS7 was eightfold higher than in the wild-type strain. Lysine supplementation increased 41% the PA production in WGS7 compared to 7% in the wild type. Similarly, serine supplementation increased the PA titre 36% in WGS7 compared to 12% in the wild type. Alanine addition did not improve PA production in WGS7. Such results suggest a clear involvement of lysine and serine on PA production in WGS7. It is possible, while highly speculative, that the ABC polar amino acid transporter may be involved in lysine and serine transport. Lysine decarboxylation is a well reported acid tolerance mechanism [41–43], which has clearly improved in WGS7 relative to the control strain. In contrast, the involvement of serine in the improved phenotype remains elusive and further investigation to elucidate its involvement is needed.

### Comparative transcriptomics

RNA-sequencing was used to compare the transcriptional profile of the strains. Our analyses identified 2406 transcribed genes, of which 76 were significantly different (*q *< 0.05). Thirteen genes were downregulated and 63 were upregulated. Based on the RNA-sequencing data, a metabolic pathway illustrating the changes in the metabolism is presented in Fig. 4. The figure includes transcripts measurements for the central carbon metabolism genes, for sugar transport, amino acid transport, the methylglyoxal pathway, the pentose phosphate pathway and the glucuronate interconversion pathway which are the main pathways showing changes in transcription.

Interestingly, WGS7 showed changes in transcription for eight ABC sugar transporters, three ABC amino acid transporters and three ABC oligopeptides transporters (Fig. 4 and Additional file 2 and Additional file 5: Table S3). The nitric oxide reductase gene (XLOC_000683) was also found to be differentially transcribed (log_2_ 1.30, *q* < 0.05). Genes belonging to the methylglyoxal pathway *ladh*, *adh*, XLOC_002338, and XLOC_002219 were also significantly transcribed in WGS7 (Fig. 4 and Additional file 9: Table S4). Three genes involved in the pentose and glucuronate interconversion were also significantly upregulated including altronate dehydratase (*uxaA*), altronate oxidoreductase (*uxaB*), and unronate isomerase (*uxaC*). The arginine/ornithine antiporter gene was downregulated in WGS7 (log_2_ 1.35, *q* < 0.05). The flavin reductase gene (XLOC_001914) and the nitric oxide reductase gene (XLOC_000683) were also found significantly upregulated (log_2_ 1.31, *q* < 0.05 and log_2_ 1.30, *q* < 0.05, respectively). Our analysis found global changes at the transcriptional level, which clearly contributed to the improved phenotype. However, the link between the genomic changes and the transcriptomics changes remains unclear. Nonetheless, as illustrated in Fig. 4, the transcriptional changes can be linked to the improved phenotype by contributing to better nutrient transport and by creating detoxifying pathways for improved metabolism.

### Energy metabolism

As mentioned earlier, WGS7 displayed a clear growth improvement relative to the control strain. Improvement in growth rate is normally linked to better energetics [44, 45]. The mutation C1487806T was found in the Cytochrome C biogenesis gene (Table 2). Cytochrome C has been reported to be abundant under anaerobic culture conditions in *E. coli* [46] and has been associated with anaerobic electron transport through nitrate reductase activity [3, 47] in the early 80 s. We found significant upregulation of the nitric oxide reductase gene (XLOC_000683); however, its involvement is unclear. We speculate that this mutation may play a role in intracellular pH control or stress response as there is a link between nitrate degradation and ammonia; the source of NO is, however, elusive given the anaerobic conditions. A report suggests that the lactate–pyruvate reaction requires an electron acceptor [3]. Other authors have suggested that cytochrome C can act as a natural electron transporter in the lactate–pyruvate reaction [48, 49]. To test this hypothesis, we performed serum bottle fermentations on CDM with 50 mM of exogenous lactate. The exogenous addition of lactate increased PA production by 125% in the WGS7 strain while only 66% in the wild type.

An electron transport system in *P. acidipropionici* is also responsible for the reduction of fumarate in the Wood-Werkman Cycle [3]. Flavin, as flavin adenine dinucleotide (FAD), has been suggested to play a role in the fumarate reductase enzyme [50]. Transcriptomics analyses showed upregulation of the flavin reductase gene (XLOC_001914). This overexpression suggests that the fumarate–succinate is contributing to the increase in PA production because it is associated with ATP generation [50]. To evaluate our hypothesis, we supplemented CDM cultures with 10 mM of fumarate. The addition of fumarate increased PA production by twofold. The fact that PA production in the wild-type was not improved by the exogenous addition of fumarate strengthens our hypothesis of a probable improvement in the electron transport chain in the fumarate–succinate reaction (Additional file 8: Figure S3).

Our transcriptomics study suggests upregulation of genes involved in the methylglyoxal pathway. The methylglyoxal pathway converts glyceraldehyde-3-phosphate into methylglyoxal [51], which generates d-lactate or l-lactate. The methylglyoxal reductase and aldehyde dehydrogenase convert methylglyoxal into lactaldehyde and produce l-lactate. If methylglyoxal enters the glyoxylate pathway, methylglyoxal is converted into d-lactate via lactoylglutathione. Lactate can be converted into pyruvate. The methylglyoxal pathway is activated under high carbon concentrations [13]. The increased sucrose uptake rate in WGS7 is likely to have triggered the methylglyoxal pathway (Figs. 2c and 4), a transient low-energy bypass of the lower Embden–Meyerhof–Parnas pathway during abundant carbon source concentrations [13]. Others have suggested that the abundance of intracellular fructose 1,6-diphosphate, glycerate-3-phosphate, or dihydroxyacetone phosphate can activate the methylglyoxal pathway [13]. In agreement with that, WGS7 showed an increased concentration of the dihydroxyacetone phosphate metabolite (Fig. 4; Additional file 7: Figure S1). Using the methylglyoxal pathway, cells have a mechanism to control the rate of energy generation relative to the overall catabolic rate.

### Economic evaluation of WGS7

**Fig. 5:**
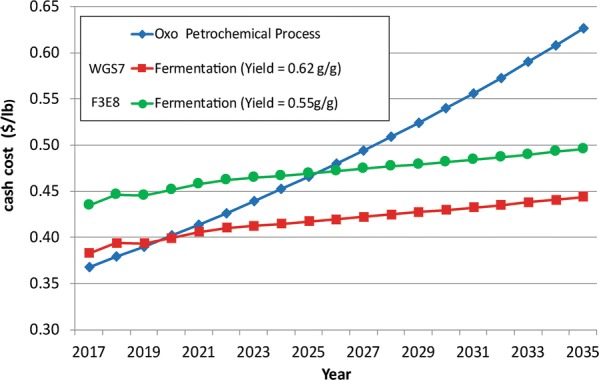
Cash cost forecast for competing PA routes using WGS7 and F3E8 strains

Prior work has shown that fermentation yield has a substantial impact on the overall economics of biological production of PA [1]. Similarly, Liu et al. [2] have suggested that PA concentration and productivity is critical to achieve a commercially viable bioprocess. The process economics model described in [1] was updated to reflect a yield improvement from 0.55 to 0.62 g/g as obtained by WGS7. Data for the Techno-economic analysis were obtained from the data available at Dow AgroSciences and the model used is described in [1]. The model predicts cost of PA production based on the current prices. As shown in Fig. 5, the yield improvement was more than a 15% cash cost reduction in 2017 and significantly improved the cost-competitiveness being 39% below the current market value of PA, suggesting commercially viable margin. The net cash costs for PA from the WGS7 process were modelled to be ~ $0.38/lb. Most importantly, the yield improvement with WGS7 reduces the time at which the fermentation process becomes economically competitive with the “oxo” process from the year 2025 to the year 2020.

## Conclusions

*Propionibacteria* are attractive biocatalysts for the biological production of C3 chemicals. Using GS, we obtained a strain capable of competitively producing PA at 170 kta scale. The fermentation yield obtained resulted in a 15% reduction in cash cost for bio-derived PA which significantly improves the cost-competitiveness with the oxo-processes. Multiomics comparison revealed genomic mutations possibly responsible for the improved phenotype. Notably, as indicated by RNA-sequencing and metabolomics, an increase in the sucrose uptake rate and an increased in amino acid uptake rate were linked to the new phenotype.

## Additional files

**Additional file 1: Table S1.** Parameters of the fermentations in serum bottles for some strains used in this study.

**Additional file 2.** Complete list of differentially expressed genes in transcriptomics.

**Additional file 3.** Supplementary information for kinetic modelling. **Table S5.** Kinetic model parameters. **Table S6.** Parameter of the 2-L fermentation with *P. acidipropionici* WGS7 in a Batch or Fed-Batch culture.

**Additional file 4: Figure S2.** Variants detected in the new genome *P. acidipropionici* WGS7 taking as a reference genome *P. acidipropionici* ATCC 55737.

**Additional file 5: Table S3.** Variants and copy number variation found in the new strain *P. acidipropionici* WGS7 using as reference strain *P. acidipropionici* ATCC 55737.

**Additional file 6.** Multiple genome alignments of mutations in WGS7 coming from a parental strain. **Figure S4.** Multiple genome alignment of the genomic regions 50 bp before and 50 bp after the mutation G1917729A was found in WGS7. ATCC55737 *P. acidipropionici* ATCC 55737; WGS7 *P. acidipropionici* WGS7; ATCC4875 *P. acidipropionici* ATCC 4875; ATCC4965 *P. acidipropionici* ATCC 4965. Position 51 indicates the mutation G1917729A in WGS7 and similarity with the other wild-type strains. This genomic region was not found in *P. jensenii* ATCC 9617 and *P. intermedium* ATCC 14072. **Figure S5.** Multiple genome alignment of the genomic region 50 bp before and 50 bp after the mutation A3335969G was found in WGS7. ATCC55737 *P. acidipropionici* ATCC 55737; WGS7 *P. acidipropionici* WGS7; ATCC4875 *P. acidipropionici* ATCC 4875; ATCC4965 *P. acidipropionici* ATCC 4965. *P. jensenii* ATCC 9617; *P. intermedium* ATCC 14072. Position 51 indicates the mutation A3335969G in WGS7 and similarity with the other wild-type strains.

**Additional file 7: Figure S1.** Correlation of intracellular metabolites between the new strain *P. acidipropionici* WGS7 and the wild-type strain *P. acidipropionici* ATCC 55737. **Table S2.** *P*-values obtained from metabolomics comparisons.

**Additional file 8: Figure S3.** Effects of exogenous addition of 50 mM of lactate (LAC), 10 mM of fumarate (FUM), 10 mM of arginine (Arg), 10 mM of lysine (Lys), 10 Mm of serine (Ser), or 10 mM of proline (Pro) in *P. acidipropionici* ATCC 55737 (light blue bars) and *P. acidipropionici* WGS7 (red bars). Fermentations were performed by duplicate serum bottle fermentations containing CDM media.

**Additional file 9: Table S4.** Legend of the main pathways associated to the PA synthesis.

## Abbreviations

PA: propionic acid
AA: acetic acid
SA: succinic acid
GS: genome shuffling
CDM: chemical defined medium
*P. acidipropionici*: *Propionibacterium acidipropionici*
PA:AA: ratio propionic acid to acetic acid
PA:SA: ratio propionic acid to succinic acid
NGS: next-generation sequencing
PAM: *Propionibacterium acidipropionici* culture media
*µ*: specific growth rate
*P*_v_: volumetric productivity
*Y*_ps_: propionic acid over glucose yield
CDS: coding sequences
*q*_s_: specific consumption rate of sucrose
*q*_p_: specific production rate of PA
*q*_AA_: specific amino acid consumption
