# Supplementary material for: Linking genotype and phenotype in an economically viable propionic acid biosynthesis process
Source: Biotechnol Biofuels. 2018 Aug 13;11:224. doi: 10.1186/s13068-018-1222-9 (PMC6090647; doi:10.1186/s13068-018-1222-9)
Supplement: Supplementary file 1 — Additional file 1: Table S1. Parameters of the fermentations in serum bottles for some strains used in this study. [file 13068_2018_1222_MOESM1_ESM.docx]

**Table S1.** Parameters of the fermentations in serum bottles for some strains used in this study

| **Strain** | **Final OD_600nm_** | **Final pH** | **PA (g/L)** | **PA/AA (g/g)** | **Yps**  **(g/g)** |
| --- | --- | --- | --- | --- | --- |
| ***P. acidipropionici* WGS.1^1^** | 19.13±0.44 | 3.82±0.01 | 8.39±0.26 | 5.58±0.25 | 0.59±0.01 |
| ***P. acidipropionici* WGS.2^1^** | 18.07±0.92 | 3.77±0.01 | 9.64±0.12 | 6.85±0.02 | 0.63±0.02 |
| ***P. acidipropionici* WGS.3^1^** | 19.65±0.21 | 3.81±0.01 | 8.67±0.05 | 6.25±0.21 | 0.58±0.02 |
| ***P. acidipropionici* WGS.4^1^** | 19.78±0.99 | 3.82±0.00 | 8.29±0.17 | 4.88±0.14 | 0.64±0.02 |
| ***P. acidipropionici* WGS.5^1^** | 19.67±0.18 | 3.82±0.02 | 9.37±0.88 | 6.30±0.91 | 0.70±0.10 |
| ***P. acidipropionici* WGS.6^1^** | 19.03±0.52 | 3.82±0.01 | 8.80±1.08 | 5.35±1.04 | 0.65±0.03 |
| ***P. acidipropionici* WGS.7^1^** | 19.06±0.20 | 3.81±0.00 | 8.67±0.27 | 5.66±0.56 | 0.75±0.05 |
| ***P. acidipropionici* WGS.8^1^** | 20.27±0.78 | 3.83±0.01 | 8.82±0.02 | 5.96±0.56 | 0.60±0.09 |
| ***P. acidipropionici* WGS.9^1^** | 19.47±0.27 | 3.83±0.01 | 8.57±0.11 | 6.01±0.06 | 0.55±0.00 |
| ***P. acidipropionici* WGS.10^1^** | 20.46±0.37 | 3.85±0.01 | 8.20±0.05 | 5.27±0.07 | 0.53±0.02 |
| ***P. acidipropionici* WGS.11^1^** | 19.69±0.58 | 3.83±0.01 | 8.81±0.98 | 5.89±0.46 | 0.57±0.08 |
| ***P. acidipropionici* WGS.12^1^** | 19.29±1.23 | 3.83±0.00 | 8.64±0.49 | 6.30±1.21 | 0.57±0.05 |
| ***P. acidipropionici* WGS.13^1^** | 18.14±0.40 | 3.87±0.01 | 8.13±0.26 | 6.43±0.96 | 0.53±0.02 |
| ***P. acidipropionici* F3E8^2^** | 16.04±0.20 | 3.92±0.01 | 6.95±0.18 | 5.26±0.29 | 0.53±0.02 |

1. New strains from GS. 2. Reference strain. PA: Propionic acid. PA/AA: Propionic acid/Acetic acid ratio. Yps: PA conversion per sugar consumed. The data represent the average of two biological replicates. The fermentation time was 96 h. The data represent the average of two biological replicates.
