# Supplementary material for: Linking genotype and phenotype in an economically viable propionic acid biosynthesis process
Source: Biotechnol Biofuels. 2018 Aug 13;11:224. doi: 10.1186/s13068-018-1222-9 (PMC6090647; doi:10.1186/s13068-018-1222-9)
Supplement: Supplementary file 3 — Additional file 3. Supplementary information for kinetic modelling. Table S5. Kinetic model parameters. Table S6. Parameter of the 2-L fermentation with P. acidipropionici WGS7 in a Batch or Fed-Batch culture. [file 13068_2018_1222_MOESM3_ESM.docx]

**Table S5.** Kinetic model parameters

| **Parameter** | **Unit** | **Value** |
| --- | --- | --- |
| *rsmax* | g/g.L | 0.0.350 |
| *Ks** | g/L | 4.220 |
| *Kpyr* | g/L | 0.170 |
| *kipa* | g/L | 7.700 |
| *kiaa* | g/L | 4.500 |
| *Yxs* | g/g | 0.820 |
| *ms* | g/g.L | 0.008 |
| *βpa* | g/g.L | 0.0010 |
| *βpyr* | g/g.L | 0.038 |
| *βaa* | g/g.L | 0.000 |
| *βsa* | g/g.L | 0.001 |
| *K1* | -- | 0.500 |
| *K2* | -- | 0.170 |
| *K3* | g/g.L | 0.041 |
| *K4* | -- | 0.105 |
| *K5* | g/g.L | 0.000 |
| *K6* | -- | 0.050 |
| *K7* | g/g.L | 0.000 |

*Ks value determined experimentally

**Table S6.** Parameter of the 2-L fermentation with *P. acidipropionici* WGS7 in a Batch or Fed-Batch culture

| **System** | **µ (1/h)** | **Yps (g/g)** | **Lactate (g/L)** | **Final PA (g/L)** |
| --- | --- | --- | --- | --- |
| **Batch** | 0.27 | 0.53 | 5.98 | 58.0 |
| **Fed-Batch** | 0.26 | 0.63 | 0 | 70 |
