# Supplementary material for: Linking genotype and phenotype in an economically viable propionic acid biosynthesis process
Source: Biotechnol Biofuels. 2018 Aug 13;11:224. doi: 10.1186/s13068-018-1222-9 (PMC6090647; doi:10.1186/s13068-018-1222-9)
Supplement: Supplementary file 4 — Additional file 4: Figure S2. Variants detected in the new genome P. acidipropionici WGS7 taking as a reference genome P. acidipropionici ATCC 55737. [file 13068_2018_1222_MOESM4_ESM.docx]

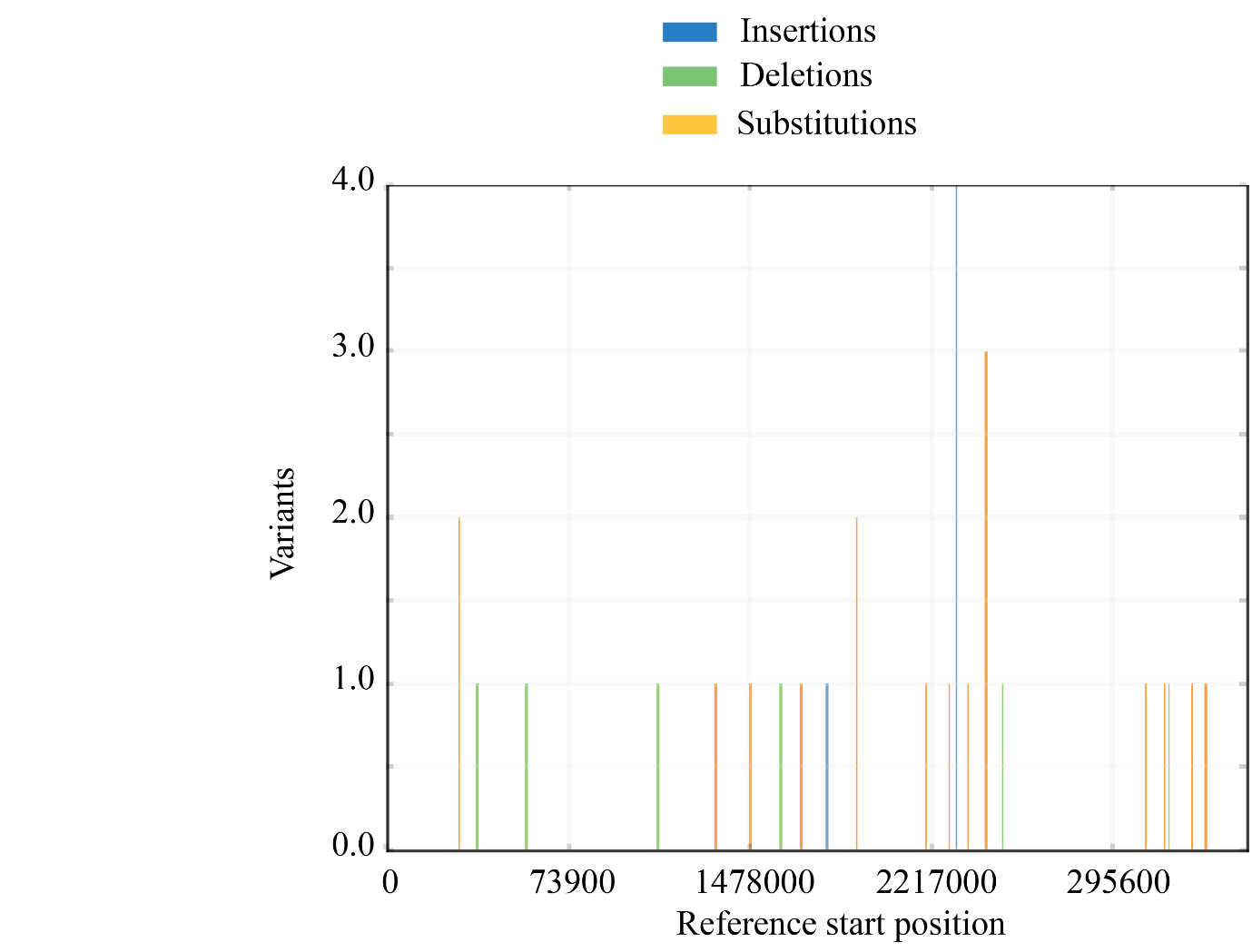


**Figure S2.** Variants detected in the new genome *P. acidipropionici* WGS7 taking as a reference genome *P. acidipropionici* ATCC 55737.
