# Supplementary material for: Linking genotype and phenotype in an economically viable propionic acid biosynthesis process
Source: Biotechnol Biofuels. 2018 Aug 13;11:224. doi: 10.1186/s13068-018-1222-9 (PMC6090647; doi:10.1186/s13068-018-1222-9)
Supplement: Supplementary file 5 — Additional file 5: Table S3. Variants and copy number variation found in the new strain P. acidipropionici WGS7 using as reference strain P. acidipropionici ATCC 55737. [file 13068_2018_1222_MOESM5_ESM.docx]

**Table S3.** Variants and copy number variation found the in the new strain *P. acidipropionici* WGS7 using as reference strain *P. acidipropionici* ATCC 55737.

| **Genome coordinate*** |  | **Type** | **Reference** | **Alternate** | | **Gene function** | **Remarks** | |
| --- | --- | --- | --- | --- | --- | --- | --- | --- |
| 294317 |  | SNP | CC | | TT | ATP-dependent DNA helicase SCO5184 | Missense | L965F |
| 371247 |  | INDEL | A | | delA | Hypothetical protein | Frameshift | Stop |
| 567004 |  | INDEL | G | | delG | 2-dehydropantoate 2-reductase | Frameshift | Stop |
| 1105158 |  | INDEL | C | | delC | Hypothetical protein | Frameshift | Stop |
| 1344547 |  | SNP | G | | A | Cobalt-zinc-cadmium resistance protein | Missense | V13M |
| 1487806 |  | SNP | C | | T | Cytochrome c-type biogenesis protein CcdA (DsbD analog) | Missense | R134Q |
| 1605833 |  | INDEL | G | | delG | Putative integral membrane protein | - | Intergenic |
| 1685121 |  | SNP | C | | T | Hypothetical protein | - | Intergenic |
| 1799226 |  | INDEL | - | | insG | Conserved membrane protein, putative permease | - | Intergenic |
| 1913841 |  | SNP | G | | T | Hypothetical protein | Silent | No Amino Acid change |
| 1917729** |  | SNP | G | | A | Multiple sugar ABC transporter, substrate-binding protein | - | Promoter |
| 2190342 |  | SNP | C | | T | Myo-inositol 2-dehydrogenase | Silent | No Amino Acid change |
|  | | Continued table S3 | | | | | | |
| 2293187 |  | SNP | C | | T | Amino acid ABC transporter, ATP-binding protein | Silent | No Amino Acid change |
| 2312506 |  | INDEL | - | | insCCAC | Hypothetical protein | - | Intergenic |
| 2360492 |  | SNP | C | | T | UDP-glucose 4-epimerase | Missense | H155Y |
| 2440725 |  | SNP | G | | C | Hypothetical protein | Silent | No Amino Acid change |
| 2440740 |  | SNP | G | | A | Hypothetical protein | Silent | No Amino Acid change |
| 2440971 |  | SNP | A | | C | Hypothetical protein | Silent | No Amino Acid change |
| 2505914 |  | INDEL | G | | delG | Ribose ABC transport system, ATP-binding protein RbsA | - | Intergenic |
| 3092919 |  | SNP | C | | G | Chromosome segregation ATPases | Missense | R127P |
| 3167868 |  | SNP | C | | G | Hypothetical protein | Missense | M1V |
| 3184231 |  | SNP | T | | G | Hypothetical protein | - | Intergenic |
| 3279129 |  | SNP | G | | A | Hypothetical protein | Missense | R62Q |
| 3335969*** |  | SNP | A | | G | LSU rRNA | - | - |

SNP: single nucleotide polymorphism; INDEL: insertion or deletion; del: deletion; ins: insertion; * Genome coordinate in *P. acidipropionici* ATCC 55737. ** The origin of this mutation can be either *P. acicipropionici* ATCC 4875 or *P. acicipropionici* ATCC 4965. *** The origin of this mutation can be from any of the *Propionibacterium* spp. strains used in this study. Intergenic: Mutation found between two genes. Stop: Incomplete expression. Promoter: Mutation found in the promoter of the gene.
