# Supplementary material for: Linking genotype and phenotype in an economically viable propionic acid biosynthesis process
Source: Biotechnol Biofuels. 2018 Aug 13;11:224. doi: 10.1186/s13068-018-1222-9 (PMC6090647; doi:10.1186/s13068-018-1222-9)
Supplement: Supplementary file 6 — Additional file 6. Multiple genome alignments of mutations in WGS7 coming from a parental strain. Figure S4. Multiple genome alignment of the genomic regions 50 bp before and 50 bp after the mutation G1917729A was found in WGS7. ATCC55737 P. acidipropionici ATCC 55737; WGS7 P. acidipropionici WGS7; ATCC4875 P. acidipropionici ATCC 4875; ATCC4965 P. acidipropionici ATCC 4965. Position 51 indicates the mutation G1917729A in WGS7 and similarity with the other wild-type strains. This genomic region was not found in P. jensenii ATCC 9617 and P. intermedium ATCC 14072. Figure S5. Multiple genome alignment of the genomic region 50 bp before and 50 bp after the mutation A3335969G was found in WGS7. ATCC55737 P. acidipropionici ATCC 55737; WGS7 P. acidipropionici WGS7; ATCC4875 P. acidipropionici ATCC 4875; ATCC4965 P. acidipropionici ATCC 4965. P. jensenii ATCC 9617; P. intermedium ATCC 14072. Position 51 indicates the mutation A3335969G in WGS7 and similarity with the other wild-type strains. [file 13068_2018_1222_MOESM6_ESM.docx]

| 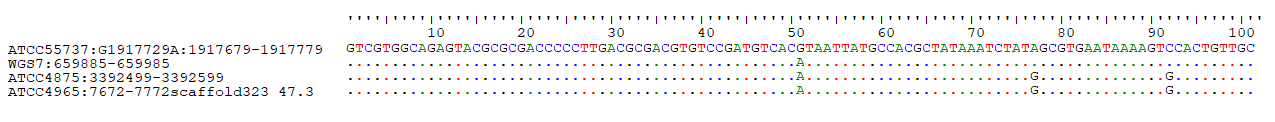 **Figure S4.** Multiple genome alignment of the genomic regions 50 bp before and 50 bp after the mutation G1917729A in WGS7. ATCC55737 *P. acidipropionici* ATCC 55737; WGS7 *P. acidipropionici* WGS7; ATCC4875 *P. acidipropionici* ATCC 4875; ATCC4965 *P. acidipropionici* ATCC 4965. Position 51 indicates the mutation G1917729A in WGS7 and similarity with the other wild-type strains. This genomic region was not found in *P. jensenii* ATCC 9617 and *P. intermedium* ATCC 14072. |
| --- |

**Figure S5.** Multiple genome alignment of the genomic region 50 bp before and 50 bp after the mutation A3335969G in WGS7. ATCC55737 *P. acidipropionici* ATCC 55737; WGS7 *P. acidipropionici* WGS7; ATCC4875 *P. acidipropionici* ATCC 4875; ATCC4965 *P. acidipropionici* ATCC 4965. *P. jensenii* ATCC 9617; *P. intermedium* ATCC 14072. Position 51 indicates the mutation A3335969G in WGS7 and similarity with the other wild-type strains.

| 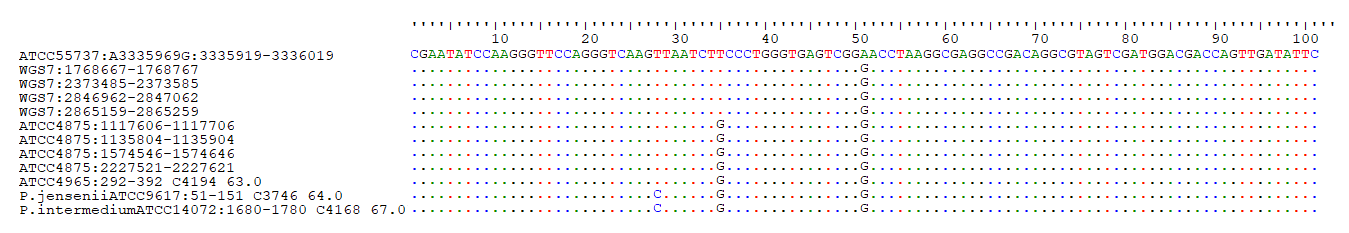 |
| --- |
