# Supplementary material for: Linking genotype and phenotype in an economically viable propionic acid biosynthesis process
Source: Biotechnol Biofuels. 2018 Aug 13;11:224. doi: 10.1186/s13068-018-1222-9 (PMC6090647; doi:10.1186/s13068-018-1222-9)
Supplement: Supplementary file 7 — Additional file 7: Figure S1. Correlation of intracellular metabolites between the new strain P. acidipropionici WGS7 and the wild-type strain P. acidipropionici ATCC 55737. Table S2. P-values obtained from metabolomics comparisons. [file 13068_2018_1222_MOESM7_ESM.docx]

**Figure S1.** Correlation of intracellular metabolites between the new strain *P. acidipropionici* WGS7 and the wild type strain *P. acidipropionici* ATCC 55737. Significant difference *p < 0.05* is indicated with (*). The intracellular metabolic data was obtained from two biological replicates. Legend of metabolites: Asp Aspartate , Glu Glutamate, Ser Serine, Gln Glutamine, Thr Threonine, Arg Arginine, Ala Alanine, GABA γ-aminobutyrate, Val Valine, Met Methionine, Phe Phenylalanine, Ile Isoleucine, Orn Ornithine, Leu Leucine, Lys Lysine, Pro Proline, 3PG 3-Phosphoglycerate, 6PG 6-Phosphogluconate, ACO Acetate, ACoA Acetyl-CoA, ADP Adenosine diphosphate, AMP Adenosine monophosphate, ATP Adenosine triphosphate, CIT Citrate, CMP Cytidine monophosphate, DHAP Dihydroxyacetone phosphate, F16DP Fructose 1-6-diphosphate, F6P Fructose-6-phosphate, Fum Fumarate, G6P Glucose-6-phosphate, GDP Guanosine diphosphate, GTP Guanosine triphosphate, NAD Nicotinamide adenine dinucleotide oxidized, NADH Nicotinamide adenine dinucleotide reduced, NADP Nicotinamide adenine dinucleotide phosphate oxidized, NADP Nicotinamide adenine dinucleotide phosphate reduced, OAA Oxaloacetate, PEP Phosphoenolpyruvate, R5P Ribose-5-phsophate, RL5P Ribulose-5-phosphate, UDP Uridine diphosphate, UDPG Uridine diphosphate glucose, UDPGA UDP-α-D-glucuronic acid, UDPNAG UDP-N-Acetyl glucosamine, UTP Uridine triphosphate, Lac Lactate, Suc Succinate, Mal Malate.

**Table S2**. *P-values* obtained from metabolomics comparisons

| **Metabolite** | ***p-value*** |
| --- | --- |
| Ser | 6.88E-07 |
| Lac | 1.23E-0.06 |
| Gly | 2.11E-0.06 |
| R5P | 0.0016 |
| DHAP | 0.0119 |
| Ala | 0.0464 |
| Arg | 0.0527 |
| PEP | 0.0739 |
| RL5P | 0.1303 |
| Suc | 0.1878 |
| 3PG | 0.1935 |
| F6P | 0.2014 |
| Gln | 0.2227 |
| Thr | 0.2338 |
| NADP | 0.3002 |
| NAD | 0.3081 |
| G6P | 0.3834 |
| GABA | 0.4056 |
| Pro | 0.4113 |
| CMP | 0.4197 |
| Phe | 0.4394 |
| NADH | 0.4479 |
| F16DP | 0.4621 |
| Leu | 0.4876 |
| Lys | 0.5229 |
| Ile | 0.5236 |
| Cys | 0.5510 |
| ACO | 0.5797 |
| OAA | 0.5945 |
| ADP | 0.5996 |
| AMP | 0.5999 |
| GDP | 0.6369 |
| UDPG | 0.6451 |
| Val | 0.6489 |
| 6PG | 0.6660 |
| NADPH | 0.6660 |
| Met | 0.6726 |
| ACoA | 0.6729 |
| Fum | 0.6904 |
| ATP | 0.7116 |
| UDPNAG | 0.7209 |

Continued Table S2

| CIT | 0.7270 |
| --- | --- |
| UTP | 0.7457 |
| Trp | 0.7500 |
| Orn | 0.7592 |
| Glu | 0.7686 |
| His | 0.7806 |
| UDPGA | 0.7869 |
| Asp | 0.7994 |
| GMP | 0.8207 |
| GTP | 0.9226 |
| UDP | 0.9541 |
| Glycol | 0.9698 |
| Mal | 0.9740 |

Legend of metabolites: see legend of Figure S1
