# Supplementary material for: Linking genotype and phenotype in an economically viable propionic acid biosynthesis process
Source: Biotechnol Biofuels. 2018 Aug 13;11:224. doi: 10.1186/s13068-018-1222-9 (PMC6090647; doi:10.1186/s13068-018-1222-9)
Supplement: Supplementary file 8 — Additional file 8: Figure S3. Effects of exogenous addition of 50 mM of lactate (LAC), 10 mM of fumarate (FUM), 10 mM of arginine (Arg), 10 mM of lysine (Lys), 10 Mm of serine (Ser), or 10 mM of proline (Pro) in P. acidipropionici ATCC 55737 (light blue bars) and P. acidipropionici WGS7 (red bars). Fermentations were performed by duplicate serum bottle fermentations containing CDM media. [file 13068_2018_1222_MOESM8_ESM.docx]

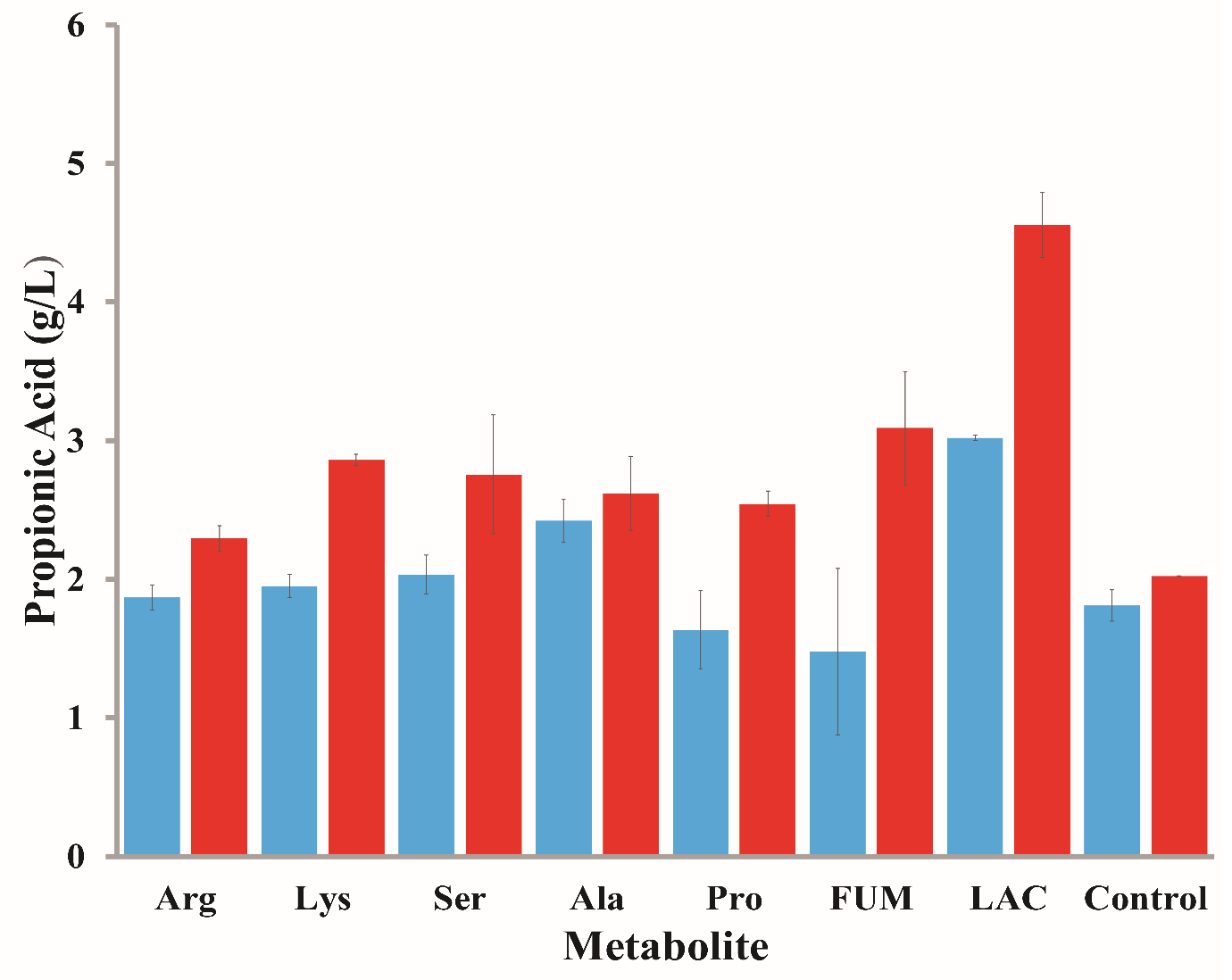


**Figure S3.** Effects of exogenous addition of 50 mM of lactate (LAC), 10 mM of fumarate (FUM), 10 mM of arginine (Arg), 10 mM of lysine (Lys), 10 Mm of serine (Ser), or 10 mM of proline (Pro) in *P. acidipropionici* ATCC 55737 (light blue bars) and *P. acidipropionici* WGS7 (red bars). Fermentations were performed by duplicate serum bottle fermentations containing CDM media. The data represent the average of two biological replicates.
