# Supplementary material for: Linking genotype and phenotype in an economically viable propionic acid biosynthesis process
Source: Biotechnol Biofuels. 2018 Aug 13;11:224. doi: 10.1186/s13068-018-1222-9 (PMC6090647; doi:10.1186/s13068-018-1222-9)
Supplement: Supplementary file 9 — Additional file 9: Table S4. Legend of the main pathways associated to the PA synthesis. [file 13068_2018_1222_MOESM9_ESM.docx]

| **Table S4**. Legend of the main pathways associated to the PA synthesis | | | |
| --- | --- | --- | --- |
| Enzyme name | EC number | Abbreviation | gene_id |
| Sucrose phosphorylase | (2.4.1.7) | - | XLOC_001439 |
| Fructokinase | (EC 2.7.1.4) | fruK | XLOC_000734 |
| Glucose-6-phosphate isomerase | (EC 5.3.1.9) | pgi | XLOC_001045 |
| 6-phosphofructokinase | (EC 2.7.1.11) | pfkA | XLOC_000890 |
| 6-phosphofructokinase, fungal/animal type | (EC 2.7.1.11) | pfkA | XLOC_000739 |
| Fructose-bisphosphate aldolase class II | (EC 4.1.2.13) | aldo | XLOC_001108 |
| Glyceraldehyde-3-phosphate ketol-isomerase | (EC 5.3.1.1) | tpi | XLOC_002246 |
| NAD-dependent glyceraldehyde-3-phosphate dehydrogenase | (EC 1.2.1.12) | gap | XLOC_001190 |
| NAD-dependent glyceraldehyde-3-phosphate dehydrogenase | (EC 1.2.1.12) | gap | XLOC_001347 |
| Enolase | (EC 4.2.1.11) | eno | XLOC_000833 |
| Enolase | (EC 4.2.1.11) | eno | XLOC_001204 |
| Pyruvate kinase | (EC 2.7.1.40) | pyk | XLOC_001333 |
| NADPH-dependent methylglyoxal reductase (L-lactaldehyde dehydrogenase) | - | ladh | XLOC_001758 |
| L-lactate dehydrogenase | (EC 1.1.1.27) | ldh | XLOC_000819 |
| 2-dehydropantoate 2-reductase | (EC 1.1.1.169) | - | XLOC_002280 |
| Aldehyde dehydrogenase | (EC 1.2.1.3) | adh | XLOC_000909 |
| Glutathione S-transferase, omega | (EC 2.5.1.18) | gst | XLOC_002338 |
| Glyoxalase family protein | - | glo | XLOC_002219 |
| Predicted D-lactate dehydrogenase, Fe-S protein, FAD/FMN-containing | - | Dldh | XLOC_002758 |
| Pyruvate dehydrogenase E1 component beta subunit | (EC 1.2.4.1) | - | XLOC_002102 |
| Pyruvate dehydrogenase E1 component | (EC 1.2.4.1) | - | XLOC_001620 |
| Dihydrolipoamide acetyltransferase component of pyruvate dehydrogenase complex | (EC 2.3.1.12) | - | XLOC_002101 |
| Dihydrolipoamide dehydrogenase | (EC 1.8.1.4) | - | XLOC_001796 |
| Pyruvate oxidase [ubiquinone, cytochrome] | (EC 1.2.2.2) | - | XLOC_001687 |
| Pyruvate-flavodoxin oxidoreductase | (EC 1.2.7.-) | - | XLOC_002427 |
| Acetyl-coenzyme A synthetase | (EC 6.2.1.1) | - | XLOC_000050 |
| Acetyl-coenzyme A synthetase | (EC 6.2.1.1) | - | XLOC_000049 |
| Pyruvate carboxylase | (EC 6.4.1.1) | pyc | XLOC_000821 |
| Citrate synthase (si) | (EC 2.3.3.1) | gltA | XLOC_000747 |
| Aconitate hydratase | (EC 4.2.1.3) | acnB | XLOC_001572 |
| Isocitrate dehydrogenase [NADP] (EC 1.1.1.42); Monomeric isocitrate dehydrogenase [NADP] | (EC 1.1.1.42) | icd | XLOC_001805 |
| Gamma-aminobutyrate:alpha-ketoglutarate aminotransferase | (EC 2.6.1.19) | - | XLOC_000668 |
| Gamma-aminobutyrate:alpha-ketoglutarate aminotransferase | (EC 2.6.1.19) | - | XLOC_001290 |
| Succinate-semialdehyde dehydrogenase [NAD(P)+] | (EC 1.2.1.16) | gabD | XLOC_000669 |
| Dihydrolipoamide succinyltransferase component (E2) of 2-oxoglutarate dehydrogenase complex (EC 2.3.1.61) / 2-oxoglutarate dehydrogenase E1 component | (EC 1.2.4.2) | - | XLOC_001491 |
| 2-succinyl-5-enolpyruvyl-6-hydroxy-3-cyclohexene-1-carboxylic-acid synthase | (EC 2.2.1.9) | menD | XLOC_000048 |
| Succinyl-CoA ligase [ADP-forming] alpha chain | (EC 6.2.1.5) | sucD | XLOC_001813 |
| Succinyl-CoA ligase [ADP-forming] beta chain | (EC 6.2.1.5) | sucD | XLOC_001814 |
| Malate dehydrogenase | (EC 1.1.1.37) | mdh | XLOC_000443 |
| Succinate dehydrogenase iron-sulfur protein | (EC 1.3.99.1) | sdh | XLOC_001667 |
| Propionyl-CoA:succinate coenzyme A transferase | (EC 5.4.99.2) | suc-coa | XLOC_001066 |
| Methylmalonyl-CoA mutase |  | - | XLOC_001262 |
| Methylmalonyl-CoA:Pyruvate transcarboxylase 5S subunit | (EC 2.1.3.1) | - | XLOC_001109 |
| Methylmalonyl-CoA:Pyruvate transcarboxylase 12S subunit | (EC 2.1.3.1) | - | XLOC_001110 |
| Clusters with Methylmalonyl-CoA carboxyltransferase | (EC 2.1.3.1) | - | XLOC_001111 |
| methylmalonyl-CoA carboxyltransferase | - | - | XLOC_000885 |
| sodium pump decarboxylase gamma subunit | - | - | XLOC_000886 |
| Oxaloacetate decarboxylase beta chain | (EC 4.1.1.3) | - | XLOC_000887 |
| methylmalonyl-CoA carboxyltransferase | - | - | XLOC_000425 |
| PTS system, beta-glucoside-specific IIB component / PTS system, beta-glucoside-specific IIC component / PTS system, beta-glucoside-specific IIA component | (EC 2.7.1.69) | PTS | XLOC_001504 |
| PTS system, beta-glucoside-specific IIB component / PTS system, beta-glucoside-specific IIC component / PTS system, beta-glucoside-specific IIA component | (EC 2.7.1.69) | PTS | XLOC_000168 |
| Sucrose-6-phosphate hydrolase | (EC 3.2.1.B3) | scrB | XLOC_002182 |

Continued Table S4...

| Enzyme name | EC number | Abbreviation | gene_id |
| --- | --- | --- | --- |
| ABC transporter, sugar binding protein | - | - | XLOC_002212 |
| Sugar ABC transporter solute-binding protein | - | - | XLOC_002351 |
| Sugar ABC transporter, sugar-binding protein | - | - | XLOC_002531 |
| sugar ABC transporter ATP-binding protein | - | - | XLOC_001896 |
| sugar ABC transporter substrate-binding protein | - | - | XLOC_000337 |
| sugar ABC transporter substrate-binding protein | - | - | XLOC_001895 |
| sugar ABC transporter substrate-binding protein | - | - | XLOC_002350 |
| Multiple sugar ABC transporter, membrane-spanning permease protein MsmF | - | - | XLOC_000692 |
| Oligopeptide ABC transporter, periplasmic oligopeptide-binding protein OppA | - | - | XLOC_002339 |
| Oligopeptide ABC transporter, periplasmic oligopeptide-binding protein OppA | - | - | XLOC_002320 |
| Oligopeptide transport system permease protein OppC | - | - | XLOC_002322 |
| Amino acid ABC transporter, ATP-binding protein | - | - | XLOC_002115 |
| amino acid ABC transporter, periplasmic amino acid-binding protein, putative | - | - | XLOC_002117 |
| Amino acid ABC transporter, ATP-binding protein | - | - | XLOC_000834 |
| Uronate isomerase | (EC 5.3.1.12) | uxaC | XLOC_000922 |
| Altronate oxidoreductase | (EC 1.1.1.58) | uxaB | XLOC_000920 |
| Altronate dehydratase | (EC 4.2.1.7) | uxaA | XLOC_000921 |
| 6-phosphogluconate dehydrogenase, decarboxylating | (EC 1.1.1.44) | gnd | XLOC_002746 |
| Glucose-6-phosphate 1-dehydrogenase | (EC 1.1.1.49) | zwf | XLOC_000387 |
